# Overexpression of a major latex-like protein from wild *Arachis* (*AdMLP11*) confers tolerance to recurrent drought stress

**DOI:** 10.1590/1678-4685-GMB-2025-0151

**Published:** 2026-07-24

**Authors:** Adrien Speck, Hugo Teixeira Gomes, Mario Alfredo Passos Saraiva, Patricia Messenberg Guimaraes, Ana Cristina Miranda Brasileiro

**Affiliations:** 1 Embrapa Recursos Genéticos e Biotecnologia, Brasília, DF, Brazil.; 2 Instituto Nacional de Ciência e Tecnologia PlantStress Biotech, Brasília, DF, Brazil.

**Keywords:** ABA signaling, Arachis duranensis, dehydration memory genes, drought tolerance ‘revised-response’

## Abstract

Recurrent drought episodes, increasingly intensified by climate change, pose a growing threat to global food security by severely limiting crop productivity. Major latex-like proteins (MLPs) play crucial roles in drought tolerance, acting as regulators of stress responses. However, their involvement in adaptation to recurrent drought stress remains poorly understood. In this study, we investigated the transcriptional dynamics of *MLP* genes during repeated dehydration and rehydration cycles in *Arachis duranensis*, a tropical wild species highly resilient to drought. *In silico* expression profiling of 36 *A. duranensis MLP* genes revealed their broad involvement in recurrent drought responses, with most patterns consistent with the ‘revised-response’ category of dehydration memory genes. qRT-PCR analysis further confirmed the activation of the abscisic acid (ABA) signaling pathway during recurrent drought in *A. duranensis*. Functional characterization of the candidate memory gene *AdMLP11* in transgenic tobacco showed that its overexpression enhances tolerance to moderate and severe recurrent drought, likely through its role as a positive regulator of phytohormone-mediated defense pathways. These findings provide novel insights into the role of MLPs in transcriptional memory and drought adaptation in wild *Arachis*, highlighting *AdMLP11* as a promising target for biotechnological strategies to develop climate-resilient crops.

## Introduction

Major latex-like proteins (MLPs) are plant-specific proteins initially discovered in the latex of opium poppy (*Papaver somniferum*) and have since been identified primarily in dicots and some monocots. These tiny proteins play critical roles in plant growth and development, stress responses, and the biosynthesis of secondary metabolites (Fujita and Inui, 2021). Structurally, MLPs are characterized by an internal hydrophobic cavity that forms a ligand-binding site, strongly suggesting their involvement in the systemic transport of hydrophobic compounds over long distances through phloem and xylem vessels (Fujita *et al*., 2022). MLPs belong to the second-largest subfamily of the white birch (*Betula verrucosa*) major pollen allergen (Bet v 1) superfamily and share a common Bet v 1 structural domain with their homologs Bet v 1s and pathogenesis-related protein 10 (PR-10). Given that many members of Bet v 1s and PR-10 families are ubiquitous plant panallergens, research efforts have focused on elucidating their allergic reactions in humans, while little attention was given to MLPs (Hoffmann-Sommergruber, 2000; Sun et al., 2024).

Recent advances in genome-wide identification and functional analysis of the MLP family (Fujita and Inui, 2021) reflect an increasing interest in their broad biological functions, particularly concerning defense responses against biotic and abiotic stresses. These studies have established that the expression of *MLP* genes is induced by a range of phytohormones involved in tolerance responses to diverse environmental stressors, including pathogens, drought, chilling, and salinity. MLPs can indirectly contribute to pathogen resistance by inducing PR genes, transducing SAR signals via phloem vessels, enhancing flavonoid content, and activating ethylene (ET) and jasmonic acid (JA) signaling pathways. For instance, the involvement of MLPs in pathogen resistance was demonstrated mainly for fungi (Holmquist et al., 2021; Kang et al., 2023; Chen et al., 2024) as well as for nematodes (Martins et al., 2020), virus (Song et al., 2020), bacteria and phytoplasma (Gai et al., 2018). Furthermore, there is a growing emphasis on the role of MLPs in conferring drought tolerance by mediating abscisic acid (ABA) signaling transduction, which leads to stomatal closure and the upregulation of abiotic stress-responsive genes (Wang et al., 2016; Liu et al., 2020; Zhou et al., 2022).

Drought is one of the most critical challenges to the global food supply, severely limiting crop yields, particularly in the context of climate change, which is anticipated to increase the frequency, duration, and severity of drought episodes, thereby exacerbating their adverse effects (Yuan et al., 2024). Over the course of evolution, plants have developed a range of molecular strategies to sense and respond to drought spells, allowing their survival under water-limited environments. Current progress in elucidating drought resilience has shifted focus towards the establishment and acquisition of stress memory, an adaptive mechanism that enables plants to retain biological imprints of past stress events and thereby become better prepared to cope with similar challenges the next time (Lagiotis et al., 2023). Such an adaptive priming process enhances future plant performance and represents a key evolutionary adaptation for survival in drought-prone environments (Pissolato et al., 2024; Siddique et al., 2024).

Over recent years, our team has studied drought tolerance mechanisms in the wild species *Arachis duranensis*, which is highly resilient to drought. Through functional genomics approaches, numerous genes and proteins associated with water deficit responses and drought tolerance of *A. duranensis* plants in their natural habitats have been uncovered (Guimaraes et al., 2012; Brasileiro et al., 2015; Vinson et al., 2018; Carmo et al., 2019). These studies have primarily focused on gene expression responses to a single drought episode, which rarely occurs in nature. However, as a multifaceted stressor, drought is characterized by notable variability in intensity, duration, and spatial distribution. In addition, transcriptome analyses in various plant species have demonstrated that molecular responses to a single drought exposure differ from those triggered by repeated exposures (Ding et al., 2012; Jacques et al., 2021).

A recent transcriptomic analysis of *A. duranensis* plants experiencing recurrent drought stress revealed an *MLP34*-coding gene among the differentially expressed genes (DEGs), which is potentially involved in the transcriptional reprogramming that marks the priming phase of drought memory establishment in this wild species (NCBI BioProject PRJNA284674). Notably, this same *MLP34* gene was also identified as a differentially abundant protein (DAP) in our previous proteomic surveys of wild *Arachis* plants submitted to a single drought episode or inoculated with the root-knot nematode (RKN) *Meloidogyne arenaria* (Carmo et al., 2019; Martins et al., 2020). Furthermore, a genome-wide analysis revealed that 20 MLP-encoding genes from cultivated peanut (*A. hypogaea*) were differentially expressed in response to waterlogging and drought stress (Li et al., 2023). Collectively, these findings highlighted the involvement of *MLP* genes, particularly *MLP34* from *A. duranensis*, renamed *AdMLP11* in a subsequent study by Li and co-workers (2023), as mediators of resistance responses to both abiotic and biotic stresses in *Arachis*.

The present study investigated the *in silico* expression profiles of the 36 *MLP* genes from *A. duranensis* in response to two cycles of dehydration and rehydration, selecting five genes for further analysis by qRT-PCR. The transcription patterns of seven ABA-related marker genes indicated that ABA signaling pathways are positively regulated in *A. duranensis* plants subjected to recurrent drought stress. We also selected the *AdMLP11* gene as a candidate for functional characterization in transgenic tobacco (*Nicotiana tabacum*) plants. The effects of *AdMLP11* transgene were further evaluated in overexpressing lines (OE) subjected to repeated drought cycles under moderate and severe water-limited conditions. Our results provide the first evidence that the heterologous overexpression of an *MLP* gene from a wild drought-tolerant species promotes enhanced responses to recurrent drought events. These findings suggest that *MLP* genes may be important components in the natural resilience to drought stress in wild Arachis species, highlighting *AdMLP11* as a promising candidate gene for biotechnological applications to enhance crop drought tolerance.

## Material and Methods

### *In silico* expression patterns of *A. duranensis MLP* genes under recurrent drought stress conditions

*In silico* expression patterns of the 36 *A. duranensis MLP* genes (*AdMLP1* to *AdMLP36*) previously identified by Li et al. (2023) were evaluated in *A. duranensis* plants submitted to two cycles of dehydration and rehydration (NCBI BioProject PRJNA284674). The first cycle consisted of a five-day no-irrigation dehydration phase (D1), during which soil moisture declined to 20% of field capacity (FC), immediately followed by a two-day rehydration phase (R1) that restored soil moisture to around 70% of FC. The second cycle followed the same protocol, with the only difference being that the dehydration phase (D2) lasted six days to reach 20% FC. Raw reads were quality-checked with Fastp v0.23.2 (Chen et al., 2018) and mapped to the *A. duranensis* reference genome (GCF_000817695.3) using STAR v2.7.10a (Dobin et al., 2013). Read counts per gene were obtained with HTSeq v0.11.1 (Anders et al., 2015). Differential expression was performed with DESeq2 (Love *et al*., 2014) after correction for unwanted variation using RUVSeq v1.34.0 (Risso et al., 2014). DEGs at stages D1, R1, D2, and R2 were identified relative to control (CTR) using thresholds of |log_2_ FC| > 2 and FDR < 0.01. The log_2_ fold-change (log_2_ FC) values of *AdMLP* gene expression across stages D1, R1, D2, and R2 were used to construct a heatmap using the ComplexHeatmap R-package (Gu et al., 2016).

### qRT-PCR analysis

Total RNA samples were extracted from *A. duranensis* plants collected at the end of each phase (D1, R1, D2, and R2), as well as from the corresponding well-watered control plants (CTR), and converted into cDNA as previously described by Martins et al. (2022). Quantitative RT-PCR (qRT-PCR) reactions were conducted in three biological replicates for each sample on a QuantStudio Real-Time PCR system (Applied Biosystems, Waltham, MA, USA), according to Morgante et al. (2013) , using specific primers pairs for the 12 *A. duranensis* target genes (Table 1). No template control (NTC) samples were included as negative controls. The real-time PCR Miner web tool (http://miner.ewindup.cn/miner/) was used to estimate primer efficiency (Zhao and Fernald, 2005). The SATqPCR web tool (Rancurel et al., 2019) was used to determine the expression ratios of transcripts from D1, R1, D2, and R2 samples relative to CTR samples (Relative Quantification; RQ) using the comparative threshold cycle (2^-ΔΔCt) method and the resulting values were statistically tested (*p* < 0.05; *t*-test). The actin (*ACT1*) and ubiquitin (*UBI2*) *Arachis* genes were used as reference genes, in accordance with Morgante *et al.* (2011).

**Table 1-:** Primers used for PCR, RT-PCR, and qRT-PCR analyses in this study.

Plant species	Analysis	Gene name	Putative function	Forward primer (5’-3’)	Reverse primer (5’-3’)	Amplicon (bp)	Reference
*Arachis duranensis*	qRT-PCR	*AdMLP6*	MLP-like protein	AAATTGCAGTCCACTCACCTG	GTCCAGTGTTTGACGGAACC	151	This study
*Arachis duranensis*	qRT-PCR	*AdMLP11*	MLP-like protein	CAGCGTTGGTGGTTCTGTTA	ACAGTGGCACCTCCATTCTC	187	Martins et al., 2020
*Arachis duranensis*	qRT-PCR	*AdMLP17*	MLP-like protein	GTGAGGACTGGCAAAGCATC	TGCATGGTGAGCTTCAAGAC	178	This study
*Arachis duranensis*	qRT-PCR	*AdMLP30*	MLP-like protein	TTGGTTCTGTCAAGCACTGG	ACAATTCCACCACCACTTCC	190	Carmo et al., 2019
*Arachis duranensis*	qRT-PCR	*AdMLP35*	MLP-like protein	GCTGTGAAGACGGTGACAGA	CCATTTCACCAAGCTTCCAT	162	This study
*Arachis duranensis*	qRT-PCR	NCED	NCED1 9-cis-epoxycarotenoid dioxygenase	TTTCTCCTCCCACGCATTCC	CCAGCAGGTGGTTTTCAAGC	181	Vinson et al., 2018
*Arachis duranensis*	qRT-PCR	ABA-Hydroxylase	abscisic acid 8’-hydroxylase 4	TTTGAGATGGCTCCAAAACC	TGAAGAGGGACAGGAAATGG	185	Vinson et al., 2018
*Arachis duranensis*	qRT-PCR	ERF-RAP2	Ethylene-responsive transcription factor RAP2-12-like	CGAGGAGCTTGCAGATATGG	TTGGGTTTCCAACATCCTGT	128	This study
*Arachis duranensis*	qRT-PCR	RD29B	Low-temperature-induced 65 kDa protein	GAACGAGACTTGCCAGAGCT	TGTGGCTGCTGGTACTTGAG	82	This study
*Arachis duranensis*	qRT-PCR	RD22	BURP domain protein RD22	CTGGGTTCCCGGATCTTACT	TTGATCCCCATACATCAAACC	170	This study
*Arachis duranensis*	qRT-PCR	GRAM	GRAM domain-containing protein	AGACGCACATAATGGGGAAG	TCGAACATGTTGAGGATGGA	190	This study
*Arachis duranensis*	qRT-PCR	LEA5	Late embryogenesis abundant protein LEA5	GGACACGCAAACAAAAACGAGT	ATGTTCCTCAACCGCAAACTCG	150	This study
*Arachis* spp.	qRT-PCR	ACT1	Actin	TGGTCTCGGTTTCCTGAGTT	AATACCACTCCAAAGCAAACG	114	Morgante et al., 2011
*Arachis* spp.	qRT-PCR	UBI2	Ubiquitin	AAGCCGAAGAAGATCAAGCAC	GGTTAGCCATGAAGGTTCCAG	145	Morgante et al., 2011
*Arachis duranensis*	PCR and RT-PCR	AdMLP11	MLP-like protein	ACCTGCTGCAAAGTTCTTCC	ACAGTGGCACCTCCATTCTC	303	This study
*Nicotiana tabacum*	PCR	bar	Phosphinothricin acetyltransferase	AAACCCACGTCATGCCAGTT	CATCGAGACAAGCACGGTCA	405	Ibrahim et al., 2017
*Nicotiana tabacum*	PCR, RT-PCR and qRT-PCR	NtL25	Ribosomal protein L25	GCTAAGGTTGCCAAGGCTGTCAAG	GCACTAATACGAGGGTACTTGGGGTTT	134	Brasileiro et al., 2021
*Nicotiana tabacum*	qRT-PCR	bar	Phosphinothricin acetyltransferase	GAAGTCCAGCTGCCAGAAAC	TCTACACCCACCTGCTGAAGT	188	This study
*Nicotiana tabacum*	qRT-PCR	NtActin	Actin	CATTGGCGCTGAGAGATTCC	GCAGCTTCCATTCCGATCA	68	Han et al., 2015

***AdMLP11* overexpression in tobacco plants**

The complete coding sequence of the *AdMLP11* gene (LOC107462779; 462 bp) was synthesized by Epoch Life Science Inc. (Sugar Land, TX, USA) and cloned under the control of the *Arabidopsis thaliana* actin 2 promoter into the unique XhoI site of the binary vector pPZP-BAR (Mota et al., 2021) that contains the *bar* gene for glufosinate-ammonium herbicide resistance and the green fluorescent protein (GFP) as reporter gene. Leaf disc explants from *in vitro* tobacco (*N. tabacum* cv. Xanthi) plants were co-cultured for 48 hours with the disarmed *Agrobacterium tumefaciens* strain ‘GV3101’ harboring the binary vector pPZP-AdMLP11, according to Horsch et al. (1985). Primary transgenic plants (T0) were *in vitro* selected on media containing the herbicide and then transferred to greenhouse conditions for growth until AdMLP11-OE lines were obtained at T1 generation, as previously described (da Silva Ferreira et al., 2024). Phenotypic traits of AdMLP11-OE lines were visually monitored throughout their growth and development and compared with WT plants to identify potential pleiotropic effects resulting from transgene insertion and/or overexpression.

Seeds from OE lines at T1 generation and untransformed wild-type (WT) controls were germinated under growth chamber conditions (25 ± 2 °C, 12 hours photoperiod; 120 μmol m^-2^ s^-1^ light intensity) and selected through three sequential applications of a 200 mg/L glufosinate-ammonium solution. The transgenic status of four herbicide-resistant OE lines (OE-1, OE-2, OE-3, and OE-15) was further assessed by PCR analysis using specific primers for the *AdMLP11* and *bar* transgenes, as well as for the reference tobacco gene *NtL25*, serving as an internal control (Table 1). In addition, to evaluate the expression of the *AdMLP11* and *bar* transgenes, total RNA was extracted from leaf tissues of the four OE-lines (OE-1, OE-2, OE-3, and OE-15) and from WT plants according to the RNeasy® Mini Kit (QIAGEN) protocol. Following treatment with DNAse, total RNA was reserve-transcribed into cDNA, as previously described (da Silva Ferreira et al., 2024) and then used as the template for both semi-quantitative (RT) and quantitative (qRT) PCR analyses. qRT-PCR reactions were conducted as described above using specific primers pairs for the *AdMLP11* and *bar* transgenes with tobacco *NtL25* and *NtActin* as reference genes (Table 1). The presence of GFP fluorescence was also observed in leaves under an M205 fluorescence stereomicroscope (Leica Microsystems, Wetzlar, Germany).

### Dry-down assay (moderate drought conditions)

Seeds from three independent OE lines (OE-1, OE-2, and OE-15) and the WT control were germinated and seedlings selected by herbicide as described above. At 30 days after sowing (DAS), homogeneous plants, were individually transplanted into 300 mL pots containing autoclaved soil and kept regularly watered under growth chamber conditions. Dry-down assay was conducted essentially as described by Brasileiro et al. (2021) , starting when the plants were 60 days old and lasting for 35 days. Following a four-week acclimation period at approximately 70% FC, the three OE lines and WT plants were each divided into two groups: a control group (CTR; n=5) and a drought-stressed group (STR; n=15). Plants from the CTR group were maintained well-watered (70% FC) throughout the 35 days of the assay. In parallel, plants from the STR group underwent six repetitive dehydration/rehydration cycles, each consisting of an interruption in irrigation for five days (dehydration phase), followed by a 2-day rehydration phase during which the plants were well-irrigated and the soil water content was kept at 70% FC. Chlorophyll content was assessed at the 3^rd^ and 5^th^ days of each dehydration phase and at the end (2^nd^ day) of each rehydration phase, based on SPAD chlorophyll meter readings (SCMR), recorded from the same leaf of each individual with a SPAD meter (SPAD-502, Konica Minolta Sensing, Japan). The relative SCMR values were calculated as the difference between the SCMR of each individual under the stress condition and the average SCMR of the individuals under the well-watered condition, divided by the average SCMR of the individuals under well-watered condition, essentially as described by Leal-Bertioli et al. (2012) .

At the end of the 6th dehydration phase (before rehydration), three leaf disc samples (0.4 cm^2^) were collected per individual from STR and CTR groups for measurements of electrolyte leakage (EL) using an adapted protocol from Whitlow et al. (1992) . Briefly, samples were incubated overnight in the dark in 10 mL of distilled water, after which initial conductivity (Xi) was measured. The solution was then boiled for 15 min, cooled, and final conductivity (Xt) was recorded. Relative electrolyte leakage was calculated as (Xi / Xt) × 100.

### Drought-hardening treatment (severe drought conditions)

Seeds from three independent OE lines (OE-1, OE-2, and OE-15) and the WT control were germinated under growth chamber conditions and selected by repeated herbicide applications as described above. At 25 DAS, homogeneous plants were transferred to a hydroponic culture system consisting of a modified ice cube tray under an opaque container of approximately 4 L. Plants were distributed in each tray according to a randomized block design and supplied with the 1.5 L of both ‘Flex Vermelho’ and ‘Flex Azul’ (PlantPAR^®^; Brazil) nutrient solutions (pH of 5.8), oxygenated using an aquarium air pump. The solution was replaced weekly to maintain nutrient stability. After a one-week acclimation period in hydroponic conditions, the three OE lines and WT plants were each divided into two groups: a control group (CTR; n=2) grown in the nutrient solution throughout the treatment and a drought-stressed group (STR; n=6) that underwent four drought-hardening cycles. The assay was conducted essentially as described by Khan et al. (2021) , starting when the plants were 32 days old and lasting for 24 days. Each drought-hardening cycle involved subjecting STR plants to a dehydration phase by withdrawing the nutrient solution from the hydroponic system for 72 hours, followed by a 48-hour rehydration phase by refilling with a fresh solution. At the end of the 4th dehydration phase (before rehydration), shoots and roots were collected to assess fresh biomass and then oven-dried at 60°C for 24 hours to determine dry biomass.

## Results

### Expression profiling of *A. duranensis MLP* genes family under recurrent drought conditions

The expression patterns of the 36 MLP-family genes identified in *A. duranensis* (Li et al., 2023) were analyzed in response to recurrent drought stress, using our previously generated transcriptome data from the drought-tolerant accession K7988, which was subjected to two dehydration and rehydration cycles (NCBI BioProject PRJNA284674). *In silico* analysis revealed that 61% (22) of the *A. duranensis MLP* genes (*AdMLPs*) were regulated under recurrent drought stress, displaying distinct expression patterns and levels in response to the different phases of the assay (D1, R1, D2, or R2).

**Figure 1 -:**
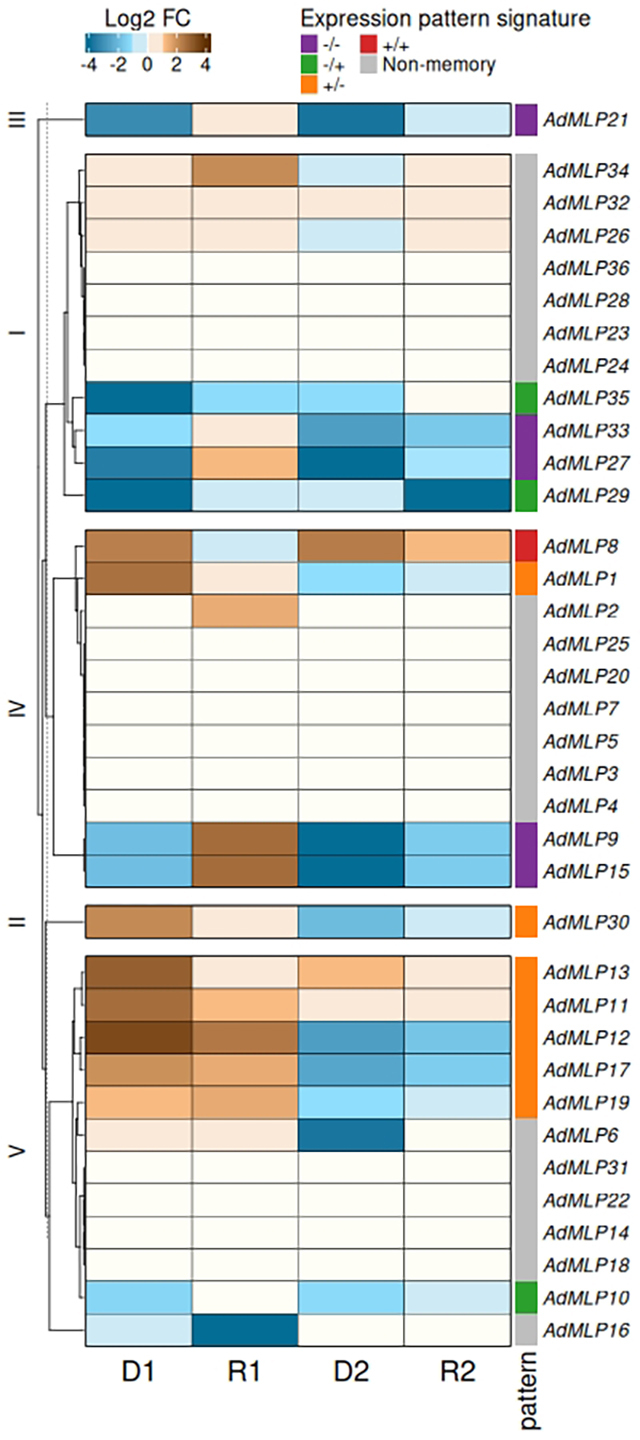
Expression profiling of *Arachis duranensis MLP* genes in response to recurrent drought stress. Heatmap showing the *in silico* expression patterns of the 36 *Arachis duranensis MLP* (*AdMLP*) genes in response to two cycles of dehydration and rehydration under recurrent drought stress. D1 and D2 correspond to the first and second dehydration phases, respectively, and R1 and R2 refer to the first and second rehydration phases, respectively. The blue-to-brown color scale represents the magnitude of differential gene expression values in log_2_ fold change (log_2_FC) at the stages D1, R1, D2, and R2 relative to the control (CTR) with a threshold of FDR < 0.01. Right column indicates the expression pattern signature of each putative memory *AdMLP* gene.

According to Jacques et al. (2021) , genes that exhibit a significant response (+ or -) to the first stress event, and that subsequently display an altered expression pattern (+ or -) in response to the subsequent stress event, are classified as memory genes that prime the plant for a more efficient response to recurrent stress. Following this concept, 16 *AdMLP* genes could be classified as memory genes and grouped into four categories based on their expression behavior in response to the initial (D1 vs. CTR) and subsequent (D2 vs. D1) drought stress phases as follows (Figure 1, Table S1): (1) seven genes that exhibited a contrasting regulation, being upregulated in D1 but producing lower transcript levels in D2 (*AdMLP1, AdMLP30, AdMLP13, AdMLP11, AdMLP12, AdMLP17,* and *AdMLP19*) designated as [+/-]; (2) five genes that were downregulated during D1 and produced transcripts at even lower levels during D2 (*AdMLP21*, *AdMLP33*, *AdMLP27*, *AdMLP9*, and *AdMLP15*) designated as [-/-]; (3) three genes that were downregulated during D1 but produced higher transcript levels in D2 (*AdMLP35, AdMLP29* and *AdMLP10*), designated as [-/+]; and (4) only one gene (*AdMLP8*) that was upregulated during D1 and produced transcripts at even higher levels during D2, designated as [+/+].

Notably, only one gene (*AdMLP6*) showed a cycle-specific response, being significantly downregulated only during D2 [=/-] (Figure 1), and therefore was considered a late-responsive, non-memory gene (Ding et al., 2013). The remaining five *AdMLP* genes (*AdMLP34, AdMLP32, AdMLP26, AdMLP2,* and *AdMLP16*) were responsive to at least one phase of the recurrent drought stress but did not show significant changes in expression in either D1 or D2.

Considering the five evolutionary groups of *AdMLP* genes (Li et al., 2023), we observed that most drought-responsive *AdMLP* genes belonging to group V were significantly upregulated during D1. In contrast, most group I genes were downregulated under this condition (Figure 1). No clear expression profile could be assigned to group IV members, and groups II and III each contained only one representative. Among the 14 *AdMLP* genes that are non-responsive to recurrent drought, distribution was uneven across groups I, IV, and V (four, six, and four members, respectively) (Figure 1).

### *A. duranensis MLP* genes are responsive to recurrent drought

**Figure 2-:**
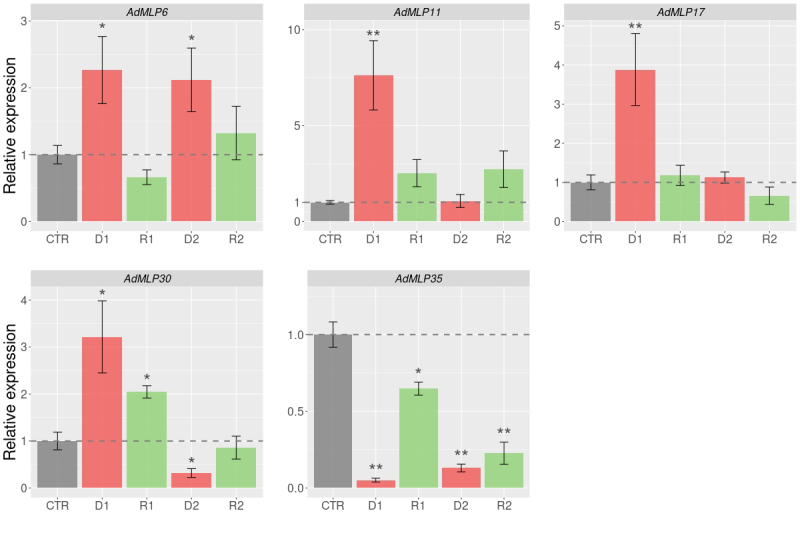
Expression analysis by qRT-PCR of five *Arachis duranensis MLP* genes in response to recurrent drought stress. Relative quantification of mRNA levels of five *Arachis duranensis MLP* (*AdMLP*) genes in response to different dehydration (D1 and D2, red) and rehydration (R1 and R2, green) phases relative to the non-stressed control (CTR, grey). The actin (*ACT1*) and ubiquitin (*UBI2*) *Arachis* genes were used as reference genes. Statistical significance was determined using Student’s *t*-test versus CTR: *p* < 0.05 (*) and *p* < 0.01 (**).

We selected five AdMLP family members for a more detailed expression analysis by qRT-PCR based on their distinct *in silico* expression patterns and their assigned evolutionary group (I, II, or V) (Figure 1). The analysis revealed that *AdMLP6* was positively regulated during both D1 and D2, reaching upregulation levels during D2 (2.1-fold), which is very similar to those observed during the first D1 stress (2.3-fold) (Figure 2; Table S2). Similarly, *AdMLP11*, *AdMLP19*, and *AdMLP30* also exhibited clear induction in response to the priming phase D1, with a significant upregulation (ranging from 3.2- to 7.6-fold) compared to the well-watered control conditions (Figure 2). However, during the second drought phase (D2), their expression dropped nearly to basal levels (*AdMLP11* and *AdMLP19*) or decreased drastically (*AdMLP30*), exhibiting, therefore, distinct expression patterns [+/-] that primarily diverged from their upregulation responses to the first phase (D1) (Figure 2). Conversely, the expression behavior of the group I representative, *AdMLP35*, was notably distinct from that of the other V and II members, showing a strong downregulation in response to both drought phases, D1 and D2, with an average decrease of 13.5-fold compared to CTR conditions (Figure 2). Overall, qRT-PCR analysis revealed that the expression of the five selected *AdMLP* genes was significantly regulated in both D1 and D2 and returned to basal levels during the first recovery phase (R1), similar to that of well-watered control plants. These basal levels were maintained after the second recovery (R2), confirming the expression profile obtained by *in silico* analysis (Figure 1).

Among the five AdMLP members analyzed, *AdMLP11* was particularly noteworthy, displaying the highest expression magnitude in response to the priming phase D1 (a 7.6-fold upregulation), which then dropped sharply to lower levels during D2 (Figure 2). *AdMLP11* was therefore selected as a candidate for further in planta functional studies.

### *A*. *duranensis* ABA-related genes are induced under recurrent drought

Defense‐related priming and responses to recurrent drought stress are orchestrated by hormone-signaling pathways, particularly ABA and JA (Avramova, 2017). Evidence also suggests that *MLP* genes may fine-tune drought stress responses by modulating ABA signaling and its downstream targets (Wang et al., 2016; Liu et al., 2020). Therefore, to investigate the involvement of ABA signaling in *A. duranensis* responses to recurrent drought stress, we analyzed the expression of seven marker genes via qRT-PCR as indicators of ABA-dependent pathway activation. These included three marker genes involved in ABA metabolism and signaling (*NCED, ABA-Hydroxylase*, and *ERF-RAP2*) and four downstream ABA-responsive genes (*RD29B*, *RD22*, *GRAM,* and *LEA5*).

**Figure 3-:**
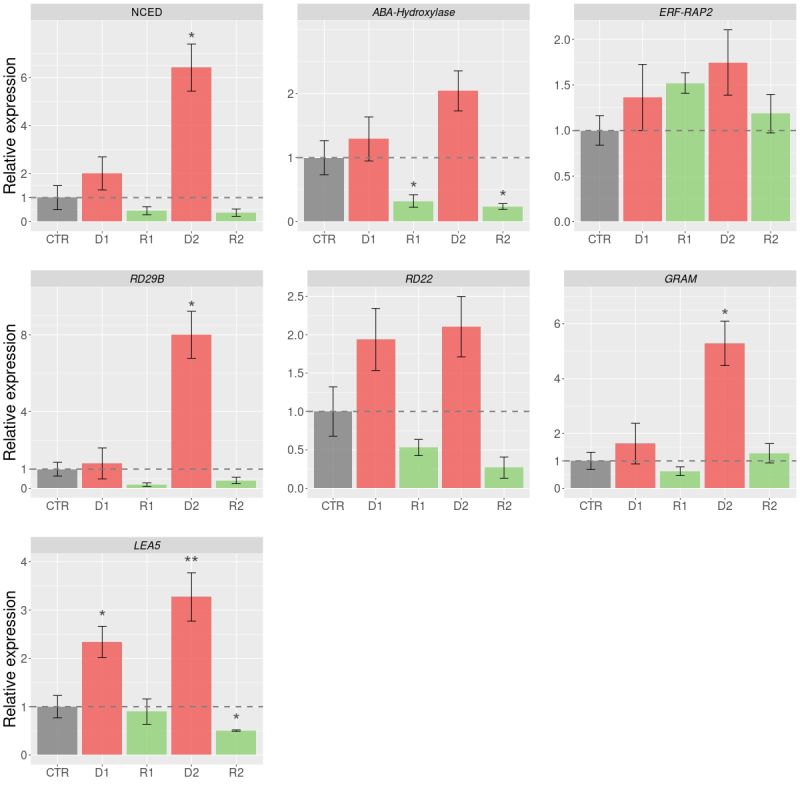
Expression analysis by qRT-PCR of seven *Arachis duranensis* ABA-related marker genes in response to recurrent drought stress. Relative quantification of mRNA levels of seven *Arachis duranensis* ABA-related marker genes in response to different dehydration (D1 and D2, red) and rehydration (R1 and R2, green) phases relative to the non-stressed control (CTR, grey). The actin (*ACT1*) and ubiquitin (*UBI2*) *Arachis* genes were used as reference genes. Statistical significance was determined using Student’s *t*-test versus CTR: *p* < 0.05 (*) and *p* < 0.01 (**). *NCED*: 9-cis-epoxycarotenoid dioxygenase; *ABA-Hydroxylase*: abscisic acid 8’-hydroxylase 4; *ERF-RAP2*: Ethylene-responsive transcription factor RAP2-12-like; *RD29B*: Low-temperature-induced 65 kDa protein; *RD22*: BURP domain protein RD22; *GRAM*: GRAM domain-containing protein; *LEA5*: Late embryogenesis abundant protein LEA5.

The expression of all of these ABA-related markers was induced in response to both drought phases (D1 and D2) compared to the control (CTR) plants, confirming the activation of ABA-signaling cascades during recurrent drought stress in *A. duranensis* (Figure 3; Table S2). However, their responses differed between drought phases, with consistently higher expression levels during the second drought phase (D2), reaching up to 6.2 times higher than during the first (D1). These results demonstrate that the responsiveness of ABA-related genes in single-stressed plants differs from that triggered in double-stressed plants, and their increased expression in D2 implies enhanced endogenous ABA levels upon subsequent drought stress exposures. In addition, except for the transcription factor *ERF-RAP2*, the expression of markers declined to stable low levels during the recovery phases (R1 and R2) (Figure 3). This dynamic regulation of ABA levels and feedback mechanisms, as well as cross-talk with other signaling pathways, is important for maintaining the balance between growth and stress responses (Ma et al., 2018). The induction of ABA-related genes under recurrent drought stress conditions in *A. duranensis* confirms previous findings in other plant species, demonstrating that activation of ABA signaling is part of the transcriptional mechanisms underlying drought memory formation (Li et al., 2019; Zuo et al., 2023).

### Generation of stable transgenic tobacco lines overexpressing *AdMLP11*

**Figure 4-:**
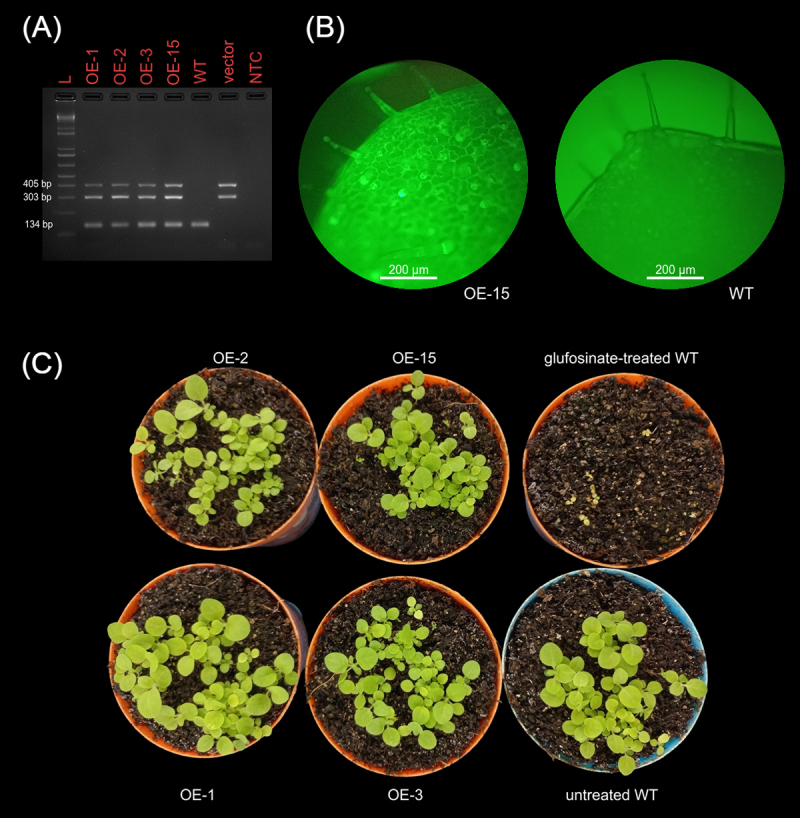
Molecular and phenotypic characterization of tobacco *AdMLP11*-overexpressing lines. (A) PCR amplification products resolved by agarose gel electrophoresis for the *bar* and *AdMLP11* transgenes (405 bp and 303 bp, respectively), and for the internal control gene *NtL25* (134 bp). Lane 1: 1 Kb DNA ladder (L); Lanes 2 to 5: tobacco AdMLP11-OE lines; Lane 6: wild-type (WT) plant, negative control; Lane 7: binary vector pPZP-AdMLP11 (vector), positive control; Lane 8: non-template control (NTC). (B) Presence of GFP fluorescence in leaves of the OE-15 line, observed under an M205 fluorescence stereomicroscope (Leica Microsystems, Wetzlar, Germany) using the Leica GFP1 filter set (excitation 480/40 nm, emission 527/30 nm). No GFP fluorescence was observed in wild-type (WT) leaves. (C) Selection of tobacco AdMLP11-OE lines by sequential application of a glufosinate-ammonium solution on T2 progeny seedlings from four OE lines (OE-1, OE-2, OE-3, and OE-15) and wild-type plants (treated WT), compared to unsprayed wild-type plants (untreated WT).

To further evaluate the effects of *AdMLP11* overexpression on recurrent drought responses, we generated 15 transgenic tobacco overexpressing (OE) lines via *Agrobacterium*-mediated leaf disc transformation. From the herbicide-resistant OE lines at the T1 generation, four (OE-1, OE-2, OE-3, and OE-15) were selected for subsequent *in planta* functional analyses. The stable transformation of these four independent OE lines was confirmed by the presence of the expected PCR products corresponding to the transgene *AdMLP11* (303 bp) and the selectable marker *bar* (405 bp), while no amplification was observed in non-transgenic WT plants (Figure 4A). The reference tobacco gene *NtL25* (134 bp), serving as an internal control, was detected in all samples. In addition, confirmation of *AdMLP11* and *bar* transgenes overexpression in these OE lines was conducted via both RT-PCR and qRT-PCR analyses, revealing slight variations in transgenes expression levels among the lines (Figure S1; Table S3).

Stable GFP expression consistently confirmed the transgenic status of PCR-positive plants at the T1 generation, with GFP fluorescence observed in the leaves of all OE lines but absent in WT controls (Figure 4B). For subsequent analyses, T2 progeny seedlings were selected for glufosinate-ammonium resistance under controlled growth chamber conditions. During the selection process, shoot tips of WT plants exhibited progressive chlorophyll degradation, ultimately leading to necrosis (Figure 4C). In contrast, OE lines recovered successfully from herbicide treatment and were regenerated into plantlets for drought assays (Figure 4C). No visually discernible differences in vegetative or reproductive traits were observed between WT plants and AdMLP11-OE lines at the T1 or T2 generations, suggesting that *AdMLP11* overexpression did not lead to visible phenotypic alterations in transgenic tobacco.

### *AdMLP11* overexpression confers tolerance to recurrent moderate drought (dry-down)

The effects of *AdMLP11* overexpression were evaluated in three independent OE lines (OE-1, OE-2, and OE-15) subjected to recurrent moderate drought cycles. Herbicide-selected T2 generation plants from these OE lines and WT controls were planted in individual pots and subjected to six consecutive drought episodes, each consisting of a 5-day dry-down phase interspersed with a 2-day rehydration phase.

**Figure 5-:**
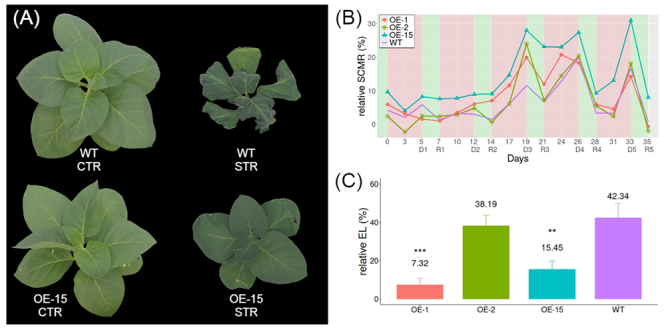
Performance of tobacco *AdMLP*-overexpressing lines during recurrent dry-down assay. (A) Aerial part phenotype of OE-15 and WT plants from the well-watered control group (CTR) and the stressed group after five cycles of dehydration and rehydration (STR). (B) Relative SPAD Chlorophyll Meter Readings (SCMR) values throughout the recurrent dry-down assay. Relative SCMR values represent the difference between SCMR under stress and the mean SCMR under well-watered conditions, normalized to the mean SCMR of well-watered plants. (C) Relative electrolyte leakage (EL) of leaves from three OE lines (OE-1, OE-2, and OE-15) and wild-type (WT) plants measured at the D5 collecting point. Data represent means ± standard deviations (n ≥ 13). Statistical significance was determined using Student’s *t*-test versus CTR plants: *p* < 0.01 (**) and *p* < 0.001 (***). ns = non-significant.

As dehydration/rehydration cycles progressed, typical visual symptoms of water deficiency became increasingly pronounced in non-transgenic WT plants, including leaf wilting, stunted growth, short stems, and smaller leaves. Moreover, these WT plants exhibited a growing inability to fully recover their pre-stress phenotype following each rehydration phase. These observations confirmed the successful imposition of recurrent drought stress as well as its cumulative detrimental effect on WT plants (Figure 5A). In contrast, although OE lines also developed water deficiency symptoms, these were markedly less severe than in WT controls. Furthermore, OE lines demonstrated a greater capacity to recover their pre-stress phenotype following rehydration compared to WT plants (Figure 5A). Throughout the assay, both well-watered OE lines and WT plants exhibited a turgid and healthy phenotype.

Relative leaf chlorophyll density was assessed daily by SCMR measurements and used as a physiological indicator for monitoring drought stress responses in tobacco plants during the assay. Both OE lines and WT plants exhibited similar patterns of change in relative SCMR values across the six dehydration/rehydration cycles. These patterns were characterized in each cycle by a progressive increase in SCMR values as soil water availability decreased, followed by a decline upon rewatering of the soil (Figure 5B). However, the amplitude of these cyclical SCMR changes in OE lines throughout the assay differed from those observed in WT plants, with generally higher values, particularly in OE-15 (Figure 5B). It is worth noting that the relative SCMR values reached both their average maximum and minimum at the end of the assay, corresponding, respectively, to the D5 point for OE-15 and the R5 point for the other OE lines and WT plants.

Another physiological index, cell membrane damage, determined by EL, was measured at the final dehydration point (D5) in both OE lines and WT plants. Water loss leads to osmotic imbalance and oxidative stress via ROS (reactive oxygen species) in plants, which compromise membrane integrity, causing ion (electrolyte) leakage from cells (Blum and Ebercon, 1981). In response to recurrent drought stress, we observed a decrease in average EL values across all three OE lines compared to WT plants, with statistically significant differences for OE-1 (*p* < 0.001; *t*-test) and OE-15 (*p* < 0.01; *t*-test) (Figure 5C; Table S4). These results provide strong evidence of reduced cell membrane damage in OE lines compared to WT controls, suggesting that *AdMLP11* overexpression may enhance membrane stability under repeated drought imposition, thereby contributing to improved drought tolerance in the transgenic plants.

### *AdMLP11* overexpression confers tolerance to recurrent severe drought (drought-hardening)

**Figure 6-:**
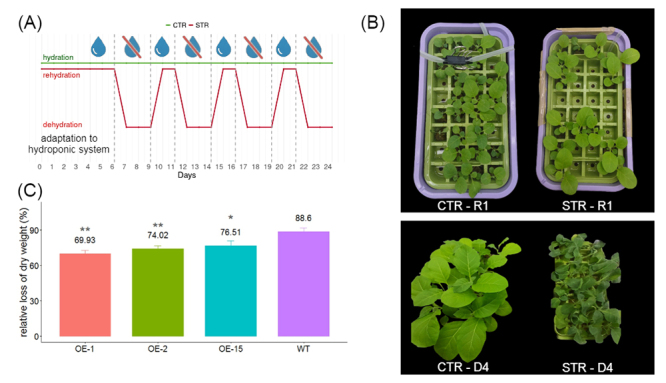
Performance of tobacco *AdMLP11*-overexpressing lines during recurrent drought-hardening treatment. (A) Schematic representation of the recurrent drought-hardening treatment: plants from the stressed group (STR, red line) were subjected to four drought-hardening cycles, each consisting of a 72-hour dehydration phase (D1 to D4) followed by a 48-hour recovery phase (R1 to R3). Plants from the well-watered control group (CTR, green line) were maintained in the nutrient solution. (B) Phenotype of plants from the well-watered control group (CTR) and the stressed group (STR) at R1 and D4 collecting points. (C) Relative dry weight of plants from three OE lines (OE-1; OE-2, and OE-15) and wild-type (WT) plants measured at the D4 collecting point. Data represent means ± standard deviations (n ≥ 6). Statistical significance was determined using the Wilcoxon test versus WT plants: *p* < 0.05 (*) and *p* < 0.01 (**).

The effects of *AdMLP11* overexpression were further evaluated in the same OE lines (OE-1, OE-2, and OE-15) under more severe recurrent drought conditions. For this, we applied a drought-hardening treatment as described by (Khan et al., 2021), with some modifications. Five cycles of drought-hardening were applied to young plants (25 DAS) using OE lines and WT controls grown under hydroponic conditions. Each cycle consisted of 72 hours of nutrient solution withdrawal (air-drying), followed by 48 hours of rewatering (Figure 6A).

As observed in the dry-down assay, WT plants exhibited gradual symptoms of water deprivation as the drought-hardening cycles progressed, with only partial recovery during the rewatering phases (Figure 6B). In contrast, OE lines exhibited milder drought symptoms and demonstrated a greater capacity to recover their pre-stress phenotype upon rehydration. Both well-watered OE lines and WT plants maintained a turgid and healthy phenotype throughout the treatment. These results validate the effectiveness of our modified drought-hardening protocol in imposing drought stress on young tobacco plants while also confirming the distinct responses of transgenic lines compared to WT plants under recurrent severe water deficit.

The drought-hardening cycles significantly reduced the total dry biomass of tobacco plants at the end of the treatment, as previously demonstrated (Khan et al., 2021). Stressed WT plants (STR group) exhibited a remarkable 88.6% decrease in biomass compared to the well-watered controls (CTR group), reflecting their impaired growth under severe drought-hardening conditions (Figure 6C). Similarly, OE lines also displayed growth retardation under stress, resulting in lower biomass with an average reduction of 73.5% compared to the CTR group. However, the biomass loss was consistently lower across all three OE lines compared to WT plants under the same stressed conditions, with statistically significant differences for OE-1 and OE-2 (*p* < 0.01; Wilcoxon test) and OE-15 (*p* < 0.05; Wilcoxon test) (Figure 6C; Table S5). These findings align with the dry-down assay results and suggest that *AdMLP11* overexpression may provide enhanced protection against dehydration, mitigating biomass loss during recurrent drought hardening stress.

## Discussion

Plants are continuously exposed to multiple concurrent environmental stresses and have evolved diverse molecular strategies to sense, respond, and adapt to them. Climate change is further intensifying these pressures as stress events become more frequent, prolonged, and severe, increasing their detrimental effects and posing novel adaptive challenges for plant survival. Throughout evolution, several *Arachis* wild species have developed tolerance to a number of environmental stresses, enabling them to survive in their stressful, natural environments. Notably, *A. duranensis* harbors drought-resilience traits and has been deployed for the identification and molecular characterization of drought-tolerance alleles through diverse functional genomics approaches (Kumar and Kirti, 2023; Massa et al., 2024). Previous studies underscore the putative role of the *MLP* gene family in drought defense responses, acting as regulators of ABA-dependent signaling pathways (Wang et al., 2016; Liu et al., 2020; Fujita and Inui, 2021; Zhou et al., 2022). In the present study, we exploited *A. duranensis* as a valuable wild drought-tolerant species to investigate the molecular mechanisms underlying the involvement of *MLP* genes in mediating tolerance responses to recurrent drought stress.

### *A. duranensis MLP* genes are involved in the response to recurrent drought stress

Comprehensive expression profiling revealed that most (61%) of the 36 *AdMLP* genes were responsive to recurrent drought stress, being either up or downregulated in primed plants (D1). The responsiveness of *AdMLP* genes to the first five days of dry-down imposition (D1) is consistent with previous studies, which showed that a single drought exposure effectively regulates the expression of *Arachis MLP* genes. Li et al. (2023) reported that ten peanut *MLP* genes were predominantly upregulated after seven days of water withholding (dry-down), but shifted to overall downregulation when stress persisted for an additional seven days (Li *et al.*, 2023). This expression pattern was observed in both waterlogging-sensitive and -resistant peanut varieties. Similarly, three proteins belonging to the *AdMLP* family were also identified as strongly modulated after just four days under dry-down conditions (Carmo et al., 2019). The broad regulation of different *MLP* genes from distinct *Arachis* species during the early stages of moderate water deficit (dry-down) may reflect transcriptional reprogramming in response to gradual soil drying, potentially allowing MLPs to mediate drought signaling from the roots to the leaves (Fujita and Inui, 2021). This regulatory pattern is not unique to *Arachis*, as *MLP* genes from tomato and poplar have also been shown to be regulated in response to moderate drought exposure simulated by PEG-induced osmotic stress (Sun et al., 2023, 2024).

However, to date, these research efforts have focused primarily on *MLP* gene responses to a single drought episode, making the present work the first to investigate MLP transcriptional dynamics in the context of recurrent drought stress.

Our findings reveal that five *MLP* genes that were downregulated during the priming phase (D1) remained repressed during the second drought phase (D2), corresponding to the [-/-] memory gene response pattern (Jacques et al., 2021). Accordingly, only one gene (*AdMLP8*) remained upregulated during both D1 and D2, displaying the [+/+] expression signature. The transcriptional [+/+] and [-/-] patterns indicate that the memory response established by these *AdMLP* genes during D1 was fully displayed in D2, and may be maintained during subsequent dehydration exposures (Liu et al., 2016).

In contrast, ten *AdMLP* genes that were significantly upregulated or downregulated during the priming phase (D1) altered their expression behavior during the subsequent drought phase (D2). These genes fall into the signature [+/-] or [-/+] memory response categories, which define the ‘revised-response’ memory behavior, as they ‘revise’ their initial transcriptional behavior upon subsequent stress exposures (Liu et al., 2014).

Evidence indicates that the activation of ‘revised-response’ memory genes with a [+/-] expression pattern is a non-specific response to a single, initial dehydration event, which includes genes responsive to other stresses that activate multiple stress-signaling pathways. However, after a recovery period, such non-specific responses are suppressed during subsequent exposures to the same stress, allowing the activation of only stress-specific genes, highlighting the key role of the revised-response in the plant stress memory mechanism. 

‘Revised response’ genes are regulated by ABA, JA, and other phytohormone signaling pathways and encode proteins involved in cellular metabolic adjustment and membrane homeostasis. The predominance of ‘revised response’ memory genes among the drought-responsive *AdMLP* genes is consistent with previous studies showing that the [+/-] or [-/+] represent the predominant patterns in *Arabidopsis*, rice, and switchgrass plants that have experienced dehydration/recovery cycles (Ding et al., 2013, 2014; Liu et al., 2014; Zhang et al., 2018).

Further expression analysis by qRT-PCR of five selected *AdMLP* genes confirmed their distinct expression patterns in D1 versus D2, regardless of their assigned evolutionary grouping. Based on these findings, we propose that *AdMLP* genes, in addition to their known roles in plant tolerance responses to single drought episodes, may also participate in recurrent drought responses and the establishment of transcriptional stress memory primarily as ‘revised response’ dehydration memory genes.

It is worth noting that the remaining 14 (40%) of *AdMLP* genes that were not responsive to recurrent drought may be involved in regulating other molecular processes. Previous studies suggest that MLP family members respond to various phytohormones, which play key roles in plant growth and development, and mediate responses to other abiotic stresses, such as cadmium exposure, salinity, and cold (Sun et al. 2024, 2025). Additionally, MLPs have been implicated in defense responses against diverse biotic challenges, including bacterial, nematode, and fungal infections (Martins et al., 2020; Fujita et al., 2021; Holmquist et al., 2021; Kang et al., 2023; Chen et al., 2024).

### ABA signaling pathway is activated by recurrent drought stress in *A. duranensis*

*Cis*-acting elements play key roles in regulating gene expression under various stress conditions by serving as binding sites for transcription factors that activate defense-related pathways. The type, distribution, and patterning of these regulatory elements within promoter sequences determine the temporal and spatial expression patterns of stress-responsive genes (Schmitz et al., 2022). The promoter regions of *MLP* genes from *A. duranensis, A. ipaënsis,* and *A. hypogaea* contain multiple stress-related cis-elements (Li et al., 2023). In particular, the abundant presence of *ABRE* (ABA-responsive element) motifs in the promoter region of *AdMLP* genes suggests that they are probably targets of ABA-mediated signaling.

In the present study, the analysis of expression profiles of genes associated with the ABA signaling pathway revealed a significant upregulation throughout the recurrent drought treatment in *A. duranensis*, with higher expression levels during the second drought event (D2) compared to the initial (D1) and a return to basal levels during recovery. Notably, the expression patterns of key ABA metabolism and signaling markers (*NCED*, *ABA-Hydroxylase*, and *ERF-RAP2*) are consistent with the transcription memory responses model proposed by Avramova (2019)  for recurrent dehydration stress. According to this model, the first dehydration stress elevates ABA levels, activating ABA signaling pathways, while during the recovery phase, ABA is no longer synthesized and the pathway becomes inactive. Upon a second dehydration exposure, ABA biosynthesis is reactivated; however, the JA‐MYC2 branch of the signaling cascade remains suppressed due to the absence of JA synthesis. This suppression seems to affect the expression of certain ‘revised-response’ [+/-] memory genes, such as some of the *AdMLP* genes here identified, that could be co-regulated by ABA and JA pathways (Fujita and Inui, 2021).

Our analysis also showed that the transcript levels of ABA-responsive genes (*RD22*, *RD29B*, *LEA5*, and *GRAM*) were upregulated under both initial (D1) and recurrent (D2) drought conditions. These marker genes have well-established roles in the molecular mechanisms and regulatory pathways that mitigate drought stress responses. In particular, *RD29B* is a memory marker gene that mediates local and systemic defense priming against drought, while coordinating plant growth fitness under stress conditions (Liu et al., 2023b). Furthermore, *LEA* genes account for 3% of memory genes exhibiting a [+/+] expression signature, which participate in responses to multiple exposures to dehydration stresses in *Arabidopsis* (Ding et al., 2013).

### *AdMLP11* overexpression confers tolerance to recurrent drought stress

As expected for a ‘revised-response’ dehydration memory gene (Ding et al., 2013), *AdMLP11* exhibited a robust magnitude of expression in response to the priming phase D1, the highest among the *AdMLP* gene family. Then, after the first recovery phase (R1), it did not respond to the subsequent dehydration phase (D2), instead maintaining its initial, pre-stressed transcription levels. The *in silico AdMLP11* expression behavior [+/-] was confirmed by qRT-PCR analysis, supporting its potential involvement in the formation of transcriptional drought memory in *A. duranensis*. Notably, *AdMLP11* was also identified as a DEG in our recent transcriptomic survey of *A. duranensis* plants undergoing two cycles of dehydration/rehydration (NCBI BioProject PRJNA284674). Therefore, *AdMLP11* was selected for *in planta* functional analysis aiming to better understand the role of *AdMLP* genes, particularly as a putative ‘revised-response’ dehydration memory gene, in the establishment of stress memory in wild *Arachis.*

Here, we demonstrated that *AdMLP11* overexpression confers enhanced drought tolerance and improved recovery capacity in transgenic tobacco plants under both moderate and severe drought conditions. In this study, moderate drought was defined as six cycles of dehydration imposed by a gradual reduction in soil water availability (dry-down or soil drying), while severe drought involved rapid water loss through air-drying (drought-hardening) by hydroponic withdrawal of the nutrient solution. Both treatments aimed to simulate scenarios in which plants experience successive drought and recovery phases, thereby allowing the assessment of plant resilience (Khan et al., 2021).

Physiological indices revealed that *AdMLP11* OE lines maintained photosynthetic efficiency, cell membrane integrity, and optimized water use across successive drought cycles, reflected by stable SCMR values, reduced electrolyte leakage, and minimized biomass loss, respectively. This improved physiological performance, compared to that of WT plants, suggests an adaptive response in OE lines, enabling sustained growth under repeated drought conditions.

Our previous proteomic surveys identified *AdMLP11* as a differentially abundant protein in wild *Arachis* species subjected to a single drought episode, as well as in response to RKN M. arenaria infection (Carmo et al., 2019; Martins et al., 2020). Further *in planta* functional validation demonstrated that *AdMLP11* overexpression in a susceptible peanut cultivar significantly reduced *M. arenaria* gall formation and egg mass production, establishing the first evidence for the involvement of an *MLP* gene in plant nematode resistance (Martins *et al.,* 2020). Taken together, these findings suggest that *AdMLP11* overexpression confers a broader protective role against both abiotic and biotic stresses, such as recurrent drought and nematode infection. Moreover, they support previous studies showing that heterologous overexpression of *MLP* genes from various plant species enhances tolerance to a broad range of abiotic and biotic stressors in different transgenic backgrounds, including tobacco, rice, and *Arabidopsis* (Wang et al., 2016; Gai et al., 2018; Liu et al., 2020; Fujita et al., 2021; Holmquist et al., 2021; Zhou et al., 2022; Liu *et al*., 2023a; Sun et al., 2025).

Despite these studies, the molecular mechanisms underlying the enhancement of stress tolerance through overexpression of *MLP* genes remain to be fully elucidated. Notwithstanding, current evidence suggests that, when overexpressed, *MLP* genes could enhance their role as positive regulators of phytohormone signaling pathways involved in defense responses against multiple stresses. This includes pathways mediated by JA, ET, and their associated crosstalk, which are linked to improved disease resistance, as well as ABA, which is associated with enhanced tolerance to environmental stressors. In line with this, the overexpression of a rice *MLP* gene (*OsMLP423*) has been shown to enhance drought and salinity tolerance, suggesting a positive regulatory role in an ABA-dependent pathway by reducing water loss, regulating ABA-responsive genes, and limiting membrane damage and ROS accumulation (Zhou et al., 2022). Moreover, its localization in both the nucleus and membrane system further suggests that MLP proteins may be involved in similar molecular mechanisms underlying abiotic stress responses in different plant species. Through constitutive activation of these transduction cascades, *MLP* overexpression may fine-tune the expression of downstream defense-related genes, ultimately triggering coordinated physiological and molecular changes that collectively promote a broader and more robust plant defense response. For instance, transgenic plants overexpressing *MLP* genes from mulberry, cotton, or zucchini exhibited enhanced resistance to pathogens, attributed to altered flavonoid levels and increased expression of defense-related genes, including *PR-2*, *PR-5*, *PDF1.2*, and glucanases (Yang et al., 2015; Gai et al., 2018; Fujita et al., 2021).

In parallel, overexpression of *MLP* genes from *Arabidopsis*, cotton, poplar, or tobacco conferred tolerance to drought, salinity, chilling, or heavy metals, mediated by orchestrated adaptive mechanisms, including modulation of ABA- and/or stress-responsive gene expression, maintenance of ROS homeostasis, regulation of proline and chlorophyll content, and mitigation of membrane damage (Chen and Dai, 2010; Wang et al., 2016; Liu et al., 2020; Liu *et al*., 2023a; Sun et al., 2025). These findings are consistent with the proposed role of Bet v 1 family proteins in stress resistance, wherein their specific three-dimensional structure enables the binding and transport of stress-related hydrophobic molecules (Fujita and Inui, 2021). Accordingly, ectopic expression of *MLP* genes may enhance the transport of key molecules involved in general defense signaling, such as phytohormones, secondary metabolites, and systemic acquired resistance (SAR) signals, thereby contributing to the alleviation of stress symptoms in transgenic plants. Nevertheless, it is worth noting that different MLP genes may confer resistance through distinct mechanisms and, in some cases, *MLP* overexpression can even reduce resistance to pathogen infection by inhibiting the expression of pathogenesis-related transcriptional factors and defense genes (He et al., 2020; Kang et al., 2023).

Building on these findings, we also hypothesize that the broad protective role conferred by *AdMLP11* overexpression against both abiotic and biotic stresses may result from its increased participation in the binding or transport of molecules associated with general stress defense mechanisms. This hypothesis is further supported by the putative role of *AdMLP11* as a ‘revised-response’ dehydration memory gene, as described here, involved in a non-specific response activated by initial exposure to drought, triggering multiple stress-signaling pathways. Similarly, the *GhMLP28* gene from cotton has also been implicated in defense responses against both abiotic (salinity) and biotic (*Verticillium dahliae*) stresses, suggesting a shared molecular basis for MLP-mediated resistance to multiple types of stress (Chen and Dai, 2010; Yang et al., 2015). However, the regulatory gene networks governing the defense responses of wild *Arachis* species to multiple, concurrent, and recurrent stresses remain complex and poorly understood. Further investigations are required to elucidate the molecular mechanisms underlying *AdMLP11*’s biological functions.

## Conclusion

Recurrent drought episodes pose a major threat to global crop productivity by disrupting key physiological processes, thereby severely limiting yields under increasingly unpredictable climatic conditions. Stress-responsive genes, such as those encoding MLPs, play a key role in enhancing drought tolerance by modulating defense pathways that drive physiological and molecular adaptations, contributing to plant resilience under water deficit conditions. The present study presents the first comprehensive analysis of *MLP* functions and transcriptional dynamics in the context of recurrent drought stress. Our transcriptomic analysis revealed that 22 out of 36 *MLP* genes in the drought-tolerant wild *A. duranensis* respond to recurrent water deficit cycles, with evidence suggesting that most act as ‘revised response’ dehydration memory genes in establishing stress memory. We found that the ABA signaling pathway is activated by recurrent drought in *A. duranensis*, with ABA-related markers showing expression patterns consistent with transcriptional memory responses. Furthermore, the overexpression of one of these memory genes, *AdMLP11,* enhanced tolerance to moderate and severe recurrent drought stress in transgenic tobacco, as evidenced by physiological indicators. However, further studies are needed to determine whether *AdMLP11*-mediated drought tolerance shares a common molecular mechanism with its previously reported role in nematode resistance. Overall, this study brings new insights into the contribution of *MLPs* to wild *Arachis* drought tolerance and highlights *AdMLP11* as a promising candidate for engineering climate-adaptive crops with enhanced drought tolerance.

## Supplementary material

The following online material is available for this article:

Figure S1 - Molecular analyses of tobacco OE lines and WT. (A) RT-PCR amplification products of the *AdMLP11* (303 bp; top) and *NtL25* (134 bp; bottom) genes visualized by agarose gel electrophoresis.

Table S1 - Differential gene expression values in log_2_ fold change (log_2_FC) at stages D1, R1, D2, and R2 relative to the control (CTR) at FDR < 0.01.

Table S2 - p-values from qRT-PCR-based Student’s t-tests for five *AdMLP* and seven *A. duranensis* ABA-related marker genes at D1, R1, D2, and R2 relative to the control (CTR).

Table S3 - p-values from qRT-PCR-based Student’s t-tests comparing four *Nicotiana tabacum* transgenic OE lines and the WT control for *AdMLP11* and *bar* gene expression.

Table S4 - p-values from Student’s t-tests comparing three *Nicotiana tabacum* transgenic OE lines and the WT control for relative electrolyte leakage (EL) measured at the D5 collecting point.

Table S5 - p-values from Wilcoxon’s tests comparing three *Nicotiana tabacum* transgenic OE lines and the WT control for relative dry weight of plants measured at the D4 collecting point.

## Data Availability

 The data used to support the findings of this study are available as on Sequence Read Archive (NCBI-SRA) database under the BioProject accession number PRJNA284674.
