# Supplementary material for: Overexpression of a major latex-like protein from wild Arachis (AdMLP11) confers tolerance to recurrent drought stress
Source: Genet Mol Biol. 2026 Jul 24;49(Suppl 3):e20250151. doi: 10.1590/1678-4685-GMB-2025-0151 (PMC13403773; doi:10.1590/1678-4685-GMB-2025-0151)
Supplement: Figure S1 - [file 1415-4757-GMB-49-s3-e20250151-s1.pdf]

Genetics and Molecular Biology
DOI: https://doi.org/10.1590/1678-4685-GMB-2025-0151

Supplementary Material to "Overexpression of a major latex-like protein from wild *Arachis* (*AdMLP11*) confers tolerance to recurrent drought stress"

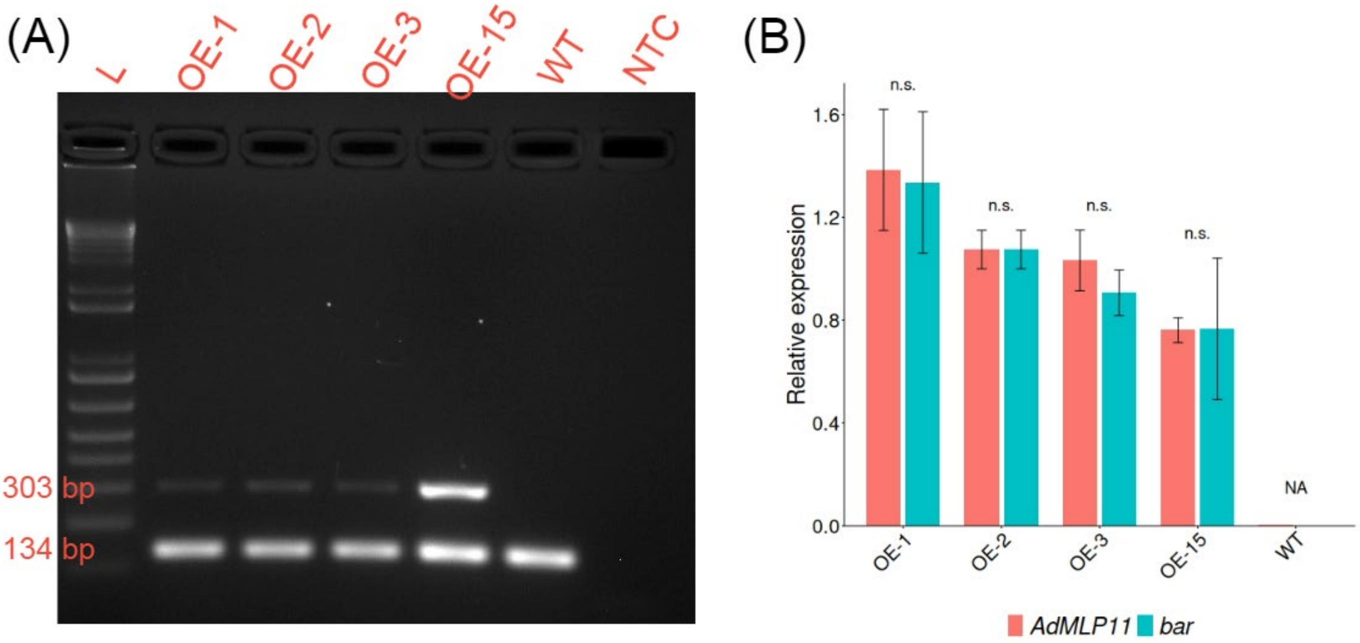

Figure S1 - Molecular analyses of tobacco OE lines and WT. (A) RT-PCR amplification products of the *AdMLP11* (303 bp; top) and *NtL25* (134 bp; bottom) genes visualized by agarose gel electrophoresis. Lane 1: 1 Kb DNA Ladder (L); Lanes 2 to 5: transgenic tobacco OE lines; Lane 6: wild-type plant negative control (WT); Lane 7: non-template control (NTC). (B) Mean normalized expression levels of the *AdMLP11* (red) and the *bar* (blue) transgenes in four tobacco OE lines normalized to the *NtL25* and *NtActin* reference genes. No statistically significant differences (n.s.) were observed among OE lines (t-test). *AdMLP11* and *bar* transgenes were not amplified (NA) in WT plants.
