# Supplementary material for: Overexpression of a major latex-like protein from wild Arachis (AdMLP11) confers tolerance to recurrent drought stress
Source: Genet Mol Biol. 2026 Jul 24;49(Suppl 3):e20250151. doi: 10.1590/1678-4685-GMB-2025-0151 (PMC13403773; doi:10.1590/1678-4685-GMB-2025-0151)
Supplement: Table S1 - [file 1415-4757-GMB-49-s3-e20250151-s2.pdf]

Genetics and Molecular Biology

DOI: https://doi.org/10.1590/1678-4685-GMB-2025-0151

Supplementary Material to "Overexpression of a major latex-like protein from wild *Arachis* (*AdMLP11*) confers tolerance to recurrent drought stress"

Table S1 - Differential gene expression values in log_2 fold change (log_2FC) at stages D1, R1, D2, and R2 relative to the control (CTR) at FDR < 0.01.

Gene name	D1	R1	D2	R2
AdMLP1	2.61	0.20	-1.14	-0.79
AdMLP2	0.00	1.49	0.00	0.00
AdMLP3	0.00	0.00	0.00	0.00
AdMLP4	0.00	0.00	0.00	0.00
AdMLP5	0.00	0.00	0.00	0.00
AdMLP6	0.78	0.38	-3.78	0.01
AdMLP7	0.00	0.00	0.00	0.00
AdMLP8	2.33	-0.48	2.37	1.25
AdMLP9	-1.91	2.72	-11.73	-1.57
AdMLP10	-1.34	0.00	-1.25	-0.51
AdMLP11	2.70	1.20	0.56	0.26
AdMLP12	3.42	2.46	-2.73	-1.76
AdMLP13	2.97	0.62	1.22	0.38
AdMLP14	0.00	0.00	0.00	0.00
AdMLP15	-1.91	2.72	-11.73	-1.57
AdMLP16	-0.79	-13.99	0.00	0.00
AdMLP17	2,00	1.50	-2.54	-1.54
AdMLP18	0.00	0.00	0.00	0.00
AdMLP19	1.22	1.52	-1.15	-0.28
AdMLP20	0.00	0.00	0.00	0.00
AdMLP21	-3.20	0.61	-3.81	-0.71
AdMLP22	0.00	0.00	0.00	0.00
AdMLP23	0.00	0.00	0.00	0.00
AdMLP24	0.00	0.00	0.00	0.00
AdMLP25	0.00	0.00	0.00	0.00
AdMLP26	0.14	0.89	-0.30	0.17
AdMLP27	-3.06	1.25	-6.11	-1.07
AdMLP28	0.00	0.00	0.00	0.00
AdMLP29	-4.47	-0.59	-0.54	-7.50
AdMLP30	2.12	0.57	-2,00	-0.71
AdMLP31	0.00	0.00	0.00	0.00
AdMLP32	0.96	0.73	0.61	0.41
AdMLP33	-1.15	0.49	-2.74	-1.65
AdMLP34	0.43	2.09	-0.43	0.61
AdMLP35	-4.54	-1.20	-1.20	0.01
AdMLP36	0.00	0.00	0.00	0.00
