# Supplementary material for: Overexpression of a major latex-like protein from wild Arachis (AdMLP11) confers tolerance to recurrent drought stress
Source: Genet Mol Biol. 2026 Jul 24;49(Suppl 3):e20250151. doi: 10.1590/1678-4685-GMB-2025-0151 (PMC13403773; doi:10.1590/1678-4685-GMB-2025-0151)
Supplement: Table S2 - [file 1415-4757-GMB-49-s3-e20250151-s3.pdf]

Genetics and Molecular Biology

DOI: https://doi.org/10.1590/1678-4685-GMB-2025-0151

Supplementary Material to "Overexpression of a major latex-like protein from wild *Arachis* (*AdMLP11*) confers tolerance to recurrent drought stress"

Table S2 - p-values from qRT-PCR-based Student's t-tests for five *AdMLP* and seven *A. duranensis* ABA-related marker genes at D1, R1, D2, and R2 relative to the control (CTR).

Gene name	CTR	D1	R1	D2	R2
AdMLP6	1	0.0347	0.1095	0.0482	0.5512
AdMLP11	1	0.0013	0.0743	0.9246	0.0543
AdMLP19	1	0.0104	0.6231	0.5898	0.3247
AdMLP30	1	0.0258	0.0163	0.0309	0.5942
AdMLP35	1	0.0013	0.0152	0.0022	0.0094
NCED	1	0.2929	0.4446	0.0255	0.3239
ABA-Hydrolase	1	0.6020	0.0467	0.0656	0.0121
ERF-RAP2	1	0.4441	0.0648	0.1277	0.5459
RD29B	1	0.9154	0.0876	0.0127	0.2603
RD22	1	0.1316	0.1795	0.1483	0.1156
GRAM	1	0.5947	0.4782	0.0159	0.5480
LEA5	1	0.0287	0.7074	0.0096	0.0435
