# Supplementary material for: Overexpression of a major latex-like protein from wild Arachis (AdMLP11) confers tolerance to recurrent drought stress
Source: Genet Mol Biol. 2026 Jul 24;49(Suppl 3):e20250151. doi: 10.1590/1678-4685-GMB-2025-0151 (PMC13403773; doi:10.1590/1678-4685-GMB-2025-0151)
Supplement: Table S3 - [file 1415-4757-GMB-49-s3-e20250151-s4.pdf]

Genetics and Molecular Biology

DOI: https://doi.org/10.1590/1678-4685-GMB-2025-0151

Supplementary Material to "Overexpression of a major latex-like protein from wild *Arachis* (*AdMLP11*) confers tolerance to recurrent drought stress"

Table S3 - p-values from qRT-PCR-based Student's t-tests comparing four *Nicotiana tabacum* transgenic OE lines and the WT control for *AdMLP11* and *bar* gene expression.

Gene	Sample	OE-1	OE-2	OE-3	OE-15	WT
AdMLP11	OE-1	1	0.3239	0.3015	0.08574	0.0310
	OE-2	NA	1	0.7703	0.0674	0.0131
	OE-3	NA	NA	1	0.1503	0.0218
	OE-15	NA	NA	NA	1	0.0126
	WT	NA	NA	NA	NA	1
bar	OE-1	1	0.4622	0.2482	0.2956	0.0359
	OE-2	NA	1	0.2865	0.4005	0.0124
	OE-3	NA	NA	1	0.611	0.0179
	OE-15	NA	NA	NA	1	0.0713
	WT	NA	NA	NA	NA	1
