# Supplementary material for: Overexpression of a major latex-like protein from wild Arachis (AdMLP11) confers tolerance to recurrent drought stress
Source: Genet Mol Biol. 2026 Jul 24;49(Suppl 3):e20250151. doi: 10.1590/1678-4685-GMB-2025-0151 (PMC13403773; doi:10.1590/1678-4685-GMB-2025-0151)
Supplement: Table S5 - [file 1415-4757-GMB-49-s3-e20250151-s6.pdf]

Genetics and Molecular Biology
DOI: https://doi.org/10.1590/1678-4685-GMB-2025-0151

Supplementary Material to "Overexpression of a major latex-like protein from wild *Arachis* (*AdMLP11*) confers tolerance to recurrent drought stress"

Table S5 - p-values from Wilcoxon's tests comparing three *Nicotiana tabacum* transgenic OE lines and the WT control for relative dry weight of plants measured at the D4 collecting point.

group1	group2	p-values	p.signif
OE-1	WT	0.0021	**
OE-2	WT	0.0086	**
OE-15	WT	0.0411	*
